# C-reactive protein level at 2 weeks following initiation of infliximab induction therapy predicts outcomes in patients with ulcerative colitis: a 3 year follow-up study

**DOI:** 10.1186/s12876-015-0333-z

**Published:** 2015-08-14

**Authors:** Ryota Iwasa, Akihiro Yamada, Koji Sono, Ryuichi Furukawa, Ken Takeuchi, Yasuo Suzuki

**Affiliations:** Department of Internal Medicine, Toho University, Sakura Medical Centre, 564-1 Shimoshizu, Sakura, Chiba, 285-8741 Japan

## Abstract

**Background:**

Poor response to anti-tumour necrosis factor biologicals like infliximab (IFX) is observed in patients with ulcerative colitis (UC), which may lead to prolonged morbidity and waste of medical resources. We aimed to look for potential biomarkers of response to IFX in patients with UC who were to undergo IFX induction therapy.

**Methods:**

Seventy-two IFX naïve UC patients with partial Mayo (pMayo) score of 4–9 received IFX infusion at weeks 0, 2 and 6 as induction therapy. The pMayo score, trough IFX and C-reactive protein (CRP) concentrations were measured. At week 14, patients who achieved a pMayo score of ≤ 2 with no individual subscore exceeding 1 were judged as responders, while patients who responded, but did not achieve a pMayo score of ≤ 2 were judged as partial responders. Likewise, patients who showed unchanged pMayo score or worsened were judged as non-responders. Patients were followed for up to 3.3 years.

**Results:**

Response, partial response and no response rates were 40.3, 33.3, and 26.4 %, respectively. CRP level at week 2 in responders was significantly lower vs partial-responders (*P* = 0.0135) or non-responders (*P* = 0.0084) in spite of similar trough IFX level. Further, the median CRP (week 2/week 0) ratio was significantly lower in patients who responded vs partial-responders or non-responders, 0.06, 0.39 and 1.00, respectively. When the cut-off value was set at 0.19 for the CRP (week 2/week 0) ratio, this ratio could predict partial-responders with 79.1 % sensitivity and 75.9 % specificity. Patients with the CRP (week 2/week 0) ratio greater than 0.19 were likely to be partial-responder, with odds ratio 10.371 (*P* < 0.0001; 95 % confidence interval 3.596–33.440).

**Conclusions:**

In this study, CRP level at week 2 following initiation of IFX induction therapy appeared to be a clinically relevant biomarker of response to IFX in UC patients.

## Background

The evolution of knowledge on the involvement of certain cytokines, notably tumour necrosis factor (TNF)-α in the immunopathogenesis of inflammatory bowel disease (IBD) has stimulated the development of anti-TNF antibodies as novel biologics for the treatment of IBD [1–3]. In deed, the efficacy of biologics like infliximab (IFX) in both Crohn’s disease (CD) and ulcerative colitis (UC) has led to a significant change in IBD treatment algorithms. However, studies on the role of TNF-α in the exacerbation of IBD have shown a greater focus on CD than on UC [3]. Nonetheless, an increased TNF-α level in the sera [4], stool [5], and colonic mucosa [6] of patients with active UC has been reported. Additionally, there are inconsistent efficacy outcomes for IFX in patients with UC refractory to corticosteroids, or to immunosuppressants [7, 8].

Serum C-reactive protein (CRP) is the most widely studied acute phase protein in inflammatory diseases and is found to have the best overall performance among laboratory markers. CRP level correlates well with disease activity in patients with CD [9, 10] and also in patients with UC [11]. The production of CRP is almost exclusively in the liver by the hepatocytes as part of an acute phase reaction in response to interleukin (IL)-6, TNF-α or IL-1β released from the site of inflammation. Additionally, a marked reduction in the level of CRP within 72 h of IFX infusion indirectly points to an effect on the cytokine profile [12].

Recently, several investigators have shown interest in understanding biomarkers of clinical response to IFX including CRP levels in CD patients [13–18]. However, their results were marred by inconsistencies; a high CRP level at baseline was thought to predict response to IFX [13, 14, 16], while a low CRP level at baseline was associated with sustained response [15]. Additionally, the reported cut-off level for CRP was different in each study. Further, in patients with CD [15, 17] or with UC [18], CRP levels drop to normal value after completion of IFX induction therapy and has been considered to predict sustained remission. However, to our knowledge, prediction of IFX responders, partial-responders or non-responders following IFX induction therapy, especially at an early stage of induction therapy has not been well investigated. With this in mind, the present study was undertaken to understand the predictive value of serum CRP at week 2 during induction therapy in IFX naïve patients with an active flare of UC. Patients were then followed up over a 3 year period to observe the association of clinical outcome with CRP level 2 weeks after the initiation of IFX induction therapy.

## Methods

### Patients

From August 2010 to June 2011, patients with moderate to severe UC refractory to corticosteroids and immunosuppressants were screened for IFX remission induction therapy [1]. Moderate to severe UC was defined by non-invasive components of Partial Mayo (pMayo) score of 4 to 9 [19]. Seventy-two eligible patients with active UC were included in this study and then were followed up to September 2013 to monitor their clinical outcomes. IFX-infusion was scheduled at weeks 0, 2 and 6. The infusion dose was set at 5 mg per kg bodyweight by using 100 mg/unit vials. For example, if a patient’s bodyweight was 56 kg, he or she was to receive a total of 3 vials (300 mg).

### Assessment of response to IFX

The clinical response to IFX was evaluated by measuring the change in the pMayo score at week 14, which was 8 weeks after the last IFX infusion. Patients who achieved a pMayo score of ≤ 2, with no individual subscore exceeding 1 was judged as responder to IFX induction therapy [20]. Patients who responded, but did not achieve a pMayo score of ≤ 2 were judged as partial-responders. Patients who showed an unchanged pMayo score or worsened were judged as non-responders. Responders including partial responders could continue to receive IFX infusion at an 8 week interval as maintenance therapy.

### Assays of serum functional IFX and CRP

Serum concentration of IFX that reflects binding capacity of IFX to biotin-labeled TNF-α was measured by a fluid-phase enzyme immunoassay reported by Yamada, et al. [21]. Assay of IFX by this method yields results similar to the monoclonal antibody-based enzyme immunoassay described by Cornillie, et al. [22]. Serum CRP concentration was measured by rate nephrometry. The lowest detectable concentration of CRP in this assay is 0.01 mg/dL, while the normal cut-off value for CRP is 0.3 mg/dL.

### Ethical considerations

Our study protocol was reviewed and approved by Ethics Committee at the Toho University Medical Centre. Regarding the potential risks of IFX therapy, prior to enrollment, patients were informed of the known, reported adverse side effects in patients with UC. Prior to IFX infusion, written informed consent was obtained from all patients. Additionally, adherence was made to the Principle of Good Clinical Practice and the Helsinki Declaration at all times.

### Statistics

When appropriate, data are presented as the median and interquantile range. Statistical analyses were done by the nonparametric Wilcoxon-Mann–Whitney test for comparing the outcomes between 2 groups. Further, qualitative data are analyzed by using the Fisher’s exact test. CRP was tested for its relevance to predict IFX partial-responders or non-responders by using receiver operating characteristic (ROC) model curves. The overall performance of the ROC analysis was determined by calculating the area under the curve (AUC). With the aid of the ROC analysis, the cut-off value with optimal sensitivity and specificity to predict partial-responders or non-responders were also calculated. All *P* values are two-tailed with the statistical significance set at *P* < 0.05, and the analyses were done by using the statistical software package (JMP, SAS Institute, Cary, NC).

## Results

### Clinical outcomes up to week 14

**Fig. 1:**
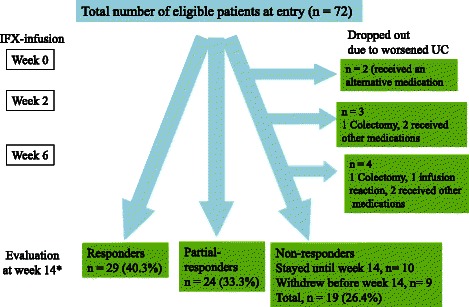
Clinical outcomes in 72 patients up to week 14. All of the 72 eligible patients had active ulcerative colitis and were infliximab naïve at entry. *Clinical response to infliximab was evaluated at week 14, patients could be divided into three subgroups: Responders (patients who achieved a partial Mayo score of ≤ 2 with no individual subscore exceeding 1); partial-responder (patients who responded, but did not achieved a partial Mayo score of ≤ 2 points); non-responders (patients in whom the partial Mayo score increased or remained unchanged relative to week 0). Only 10 of 19 patients in the non-responder sub-group were available for evaluation at week 14

The clinical response rate for IFX induction therapy following three infusions at weeks 0, 2, and 6 up to week 14 was 40.3 % (29 of 72 patients). The incidences of partial-responder and non-responder were 33.3 % (24 of 72) and 26.4 % (19 of 72), respectively. Before week 14, 9 of 19 patients in the non-responder subgroup withdrew from the study due to worsening UC (Fig. 1).

Table 1 shows patients’ main demographic variables at baseline. The median duration of UC was 4.1 years, and the median dose of IFX (mg/kg/infusion) was 5.8. The time from the initiation of IFX-induction therapy due to UC flare-up was longer in the partial-responders (*P* = 0.0255) or non-responders (*P* = 0.0072) vs responders; 107 days, 101 days and 38 days, respectively. Further, the average serum albumin level was significantly lower in the partial-responders (*P* = 0.0006) and the non-responders (*P* = 0.0022) vs responder; 4.1 g/dL, 4.0 g/dL and 4.4 g/dL, respectively. The average age was significantly shorter in the non-responder sub-group as compared with the responders, 26.1 years vs 37.5 years (*P* = 0.0315). A higher failure rate was seen for 2 or more immunosuppressants in the past, 46.2 % in the non-responders vs 17.2 % in the responders.

**Table 1:** Baseline demographic characteristics of the 72 patients with active ulcerative colitis (UC), sub-grouped as responders, partial-responders or non-responders following infliximab (IFX) induction therapy

Demography	IFX responders (*n* = 29)	IFX partial-responders (*n* = 24)	IFX non-responders (*n* = 19)	*P*_1_ Value	*P*_2_ Value
Male, number (%)	13 (44.8)	14 (58.3)	12 (63.2)	0.4117	0.2500
Age, year	37.5 [30.3–54.4]	37.6 [32.3–45.5]	26.1 [20.2–36.4]	0.9929	0.0315
Duration of UC, year	5.4 [1.8–9.3]	6.3 [2.6–10.4]	2.5 [1.0–6.1]	0.5028	0.0958
Duration (days) of active UC prior to IFX induction	38.0 [26.5–63.0]	107.0 [30.5–178.8]	101.0 [41.0–153.0]	0.0255	0.0072
Dose of infused IFX (mg/kg)	6.2 [5.3–6.7]	5.7 [5.5–6.2]	6.1 [5.3–6.6]	0.4579	0.9411
CRP (mg/dL)	1.17 [0.41–2.74]	1.28 [0.32–2.11]	0.59 [0.26–1.16]	0.8302	0.1739
Albumin (g/dL)	4.4 [4.2–4.6]	4.1 [4.0–4.3]	4.0 [3.7–4.3]	0.0006	0.0022
Haemoglobin (g/dL)	12.3 [11.4–13.6]	12.8 [11.1–13.7]	11.9 [9.9–13.4]	0.9715	0.2820
Partial Mayo Score (0–9)	7 [5–7]	7 [6–8]	7 [5–7]	0.1020	0.8872
UC location, number (%)					
Extensive	23 (79.3)	16 (66.7)	15 (79.0)	0.3579	1.0000
Left-sided	6 (20.7)	8 (33.3)	4 (21.1)	0.3579	1.0000
Corticosteroid dependent, number (%)	18 (62.1)	20 (83.3)	10 (52.6)	0.1274	0.5613
Corticosteroid refractory, number (%)	10 (34.5)	1 (4.2)	9 (47.4)	0.0075	0.5469
Concomitant medication, number (%)					
5-Aminosalicylates	27 (93.1)	23 (95.8)	18 (94.7)	1.0000	1.0000
Corticosteroids	20 (69.0)	14 (58.3)	16 (84.2)	0.5662	0.3157
Azathioprine/ Mercaptopurine	8 (27.6)	6 (25.0)	6 (31.6)	1.0000	1.0000
Previously failed ≥2 immunosuppressant, number (%)	5 (17.2)	1 (4.2)	8 (46.2)	0.2044	0.0959
Smoking status, number (%)					
Currents smoker	2 (6.9)	3 (12.5)	1 (5.3)	0.6486	1.0000
Nonsmoker	24 (82.8)	21 (87.5)	14 (73.7)	0.7153	0.4873
Past smoker	3 (10.3)	0 (0)	4 (21.1)	0.2424	0.4116

Certain values are presented as the median [interquartile range] and compared by Wilcoxon-Mann–Whitney test. Categorical variables are represented as number (%) and compared by the Fisher’s exact test. *P*1, responder vs partial-responder; *P*2, responder vs non-responder

### pMayo score, IFX and CRP levels at weeks 2 and 14

Two weeks after the first IFX infusion, we compared trough IFX, pMayo score and CRP levels in the IFX responders with the corresponding values in the IFX partial-responders and the non-responders. The pMayo score and CRP levels in responders were significantly lower than the levels in IFX partial-responders or non-responders. However, trough IFX level between the responders and partial-responders or non-responders was not significantly different (Table 2). Further, the ratio of CRP at week 2/week 0 in the responder subg-roup was significantly smaller than for IFX partial-responders or non-responders, *P* = 0.0025 and *P* < 0.0001, respectively. The same parameters and 3 individual subscores in the Mayo score at week 14 (the final clinical efficacy evaluation time point) are seen in Table 3. Differences in these parameters between the 3 groups were almost the same as shown in Table 2. The median CRP level from week 2 to week 14 in the responder sub-group had decreased from 0.07 mg/dL to 0.04 mg/dL, while in the partial-responder sub-group, CRP had increased from 0.19 mg/dL to 0.56 mg/dL. Only ten patients in the non-responder sub-group could remain in the study up to week 14.

**Table 2:** Comparison of trough serum infliximab (IFX) levels, partial Mayo Score and C-reactive protein (CRP) levels in subgroups of ulcerative colitis patients 2 weeks after the initiation of IFX infusion

Variable	IFX responders (*n* = 29)	IFX partial-responders (*n* = 24)	IFX non-responders (*n* = 19)	*P*_1_ Value	*P*_2_ Value
Trough IFX (μg/mL)	23.3 [18.9–29.7]	30.9 [17.7–42.7]	25.0 [21.6–31.5]	0.1825	0.4436
Partial Mayo Score (0–9)	1.0 [0–3.0]	5.0 [3.0–6.0]	7.0 [3.0–8.0]	<0.0001	<0.0001
CRP (mg/dL)	0.07 [0.02–0.24]	0.19 [0.08–0.67]	0.43 [0.16–2.13]	0.0135	0.0084
CRP (week 2/week 0) ratio	0.06 [0.02–0.21]	0.39 [0.08–0.97]	1.00 [0.29–1.89]	0.0025	<0.0001

Data are presented as the median [interquartile range] values and compared by Wilcoxon-Mann–Whitney test

*P*_1_, responder vs partial-responder; *P*_2_: responder vs no-responder

**Table 3:** Comparison of trough serum infliximab (IFX), C-reactive protein (CRP) levels, partial Mayo Score and subscores in subgroups of patients with ulcerative colitis at week 14 following the initiation of IFX infusion

Variable	IFX responders (*n* = 29)	IFX partial-responders (*n* = 24)	IFX non-responders (*n* = 10^a^)	*P*_1_ Value	*P*_2_ Value
Trough IFX (μg/mL)	12.4 [3.2–22.4]	9.1 [3.9–13.9]	17.6 [4.9–28.8]	0.8851	0.7274
CRP (mg/dL)	0.04 [0.01–0.07]	0.56 [0.27–0.96]	0.11 [0.02–0.50]	0.0009	0.01911
Partial Mayo Score (0–9)	0 [0–1]	4 [4–6]	6.5 [5.5–7.3]	<0.0001	<0.0001
Stool frequency (0–3)	0 [0–0]	2 [2–3]	3 [2–3]	<0.0001	<0.0001
Rectal bleeding (0–3)	0 [0–0]	1 [1–2]	2 [2–2.5]	<0.0001	<0.0001
PGA (0–3)	0 [0–0]	1 [1–1]	2 [1.5-2]	<0.0001	<0.0001

Data are presented as the median [interquartile range] values and compared by Wilcoxon-Mann–Whitney test. *P*_1_, responder vs partial-responder; *P*_2_, responder vs non-responder

*PGA* Physician’s global assessment

^a^ Only 10 of 19 patients were available for evaluation at week 14 visit, the other 9 had withdrawn due to worsened UC

### The predictive value of CRP 2 weeks after starting IFX-induction therapy

**Fig. 2:**
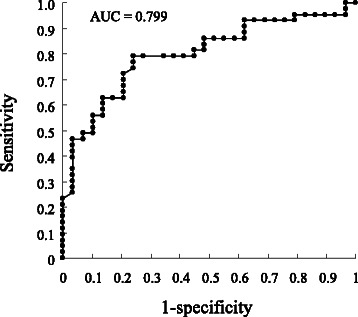
Receiver operating characteristic graphs to assess the significance of C-reactive protein (week 2/week 0) ratio for the prediction of infliximab partial-responder in patients with active ulcerative colitis undergoing infliximab-induction therapy. A cut-off value of 0.19 for C-reactive protein ratio on the receiver operating characteristic graph could predict infliximab partial-responder feature with a 79.1 % sensitivity and 75.9 % specificity

We applied a receiver operating characteristic (ROC) curve model to test the relevance of CRP (week 2/week 0) ratio to identify IFX partial-responder or non-responder feature (Table 2). A cut-off value of 0.19 (week 2/week 0 ratio) on the ROC curve could predict an IFX partial-responder feature with a 79.1 % sensitivity and 75.9 % specificity. The area under the ROC curve (AUC) which reflected the overall performance of the ROC analysis was 0.799 (Fig. 2). Patients with the CRP (week 2/week 0) ratio greater than 0.19 were likely to be partial-responder with the odds ratio 10.371 (*P* < 0.0001; 95 % confidence interval 3.596–33.440). Further, we followed the ROC model for baseline serum albumin level. If the cut-off value was set at 4.1 g/dL, it could predict an IFX partial-responder feature with 62.8 % sensitivity and 86.2 % specificity. However, baseline concentration of albumin did not appear to be a sensitive predictor of response to IFX.

### Week 2/week 0 CRP ratio, and the incidence of colectomy

**Fig. 3:**
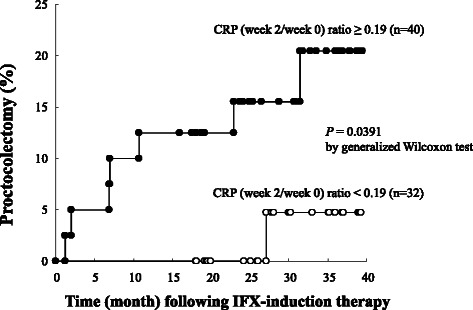
The Kaplan-Meier estimator plots for the probability of proctocolectomy in subgroups of patients with cut-off value of 0.19 C-reactive protein (week 2/week 0) ratios following the initiation of infliximab-induction therapy

We followed up the 72 patients for more than 3 years, average 39.5 months after the initiation of IFX induction therapy, focusing on the changes in the week 2/week 0 CRP ratio. The cumulative probability rates of proctocolectomy in patients with the cut-off value of CRP (week 2/week 0) ratio greater than 0.19, and patients with a lower cut-off value were 20.5 and 4.8 %, respectively (Fig. 3). Seven patients among the 40 who showed a cut-off value greater than 0.19 in the CRP (week 2/week 0) ratio had undergone proctocolectomy. Five patients were IFX non-responders and two were partial-responders. Among these 7 patients, the mean time to undergo proctocolectomy after the diagnosis of UC was 6.5 years, range 0.8 to 12.4 years. Only 1 patient among the 32 who showed a lower than 0.19 cut-off CRP (week 2/week 0) ratio had undergone proctocolectomy. This patient was responder to IFX, but discontinued IFX-maintenance therapy due to life-threatening pneumonia at 3.7 months after the initiation of IFX induction therapy.

## Discussion

In this study, we noticed that following the first IFX infusion, serum CRP level at week 2 in IFX responders significantly decreased relative to baseline, but a similar trend was not observed in non-responders. Potentially such observation should identify IFX responders at an early stage during induction therapy. Therefore, at week 2, the pMayo score was significantly smaller in the IFX responders vs partial-responders or non-responders, in spite of trough IFX level not showing any significant difference between these sub-groups. Further, the ratio of serum CRP level at week 2/week 0 appeared to be more meaningful than we had expected. Then we took advantage of this finding and applied an ROC model to see if the CRP ratio had any clinically relevant relationship with IFX responder feature or otherwise partial-responder and non-responders features. For the CRP ratio, a cut-off value of 0.19 on the ROC graph predicted IFX partial-responder feature with a 79.1 % sensitivity and 75.9 % specificity. Finally, in patients with the cut-off value of the CRP ratio being greater than 0.19, the cumulative probability of proctocolectomy was significantly higher than in patients with a lower cut-off CRP ratio during the follow-up period. Accordingly, changes in CRP at 2 weeks after the initiation of IFX induction therapy may predict or be associated with long-term clinical outcomes.

In the presented study, with respect to the clinical response to IFX-induction therapy, patients could be divided into 3 subgroups, responders, partial-responders and non-responders when evaluated at week 14. These clinical outcomes were associated with the changes in serum CRP level at week 2 during IFX-induction therapy. A marked reduction in the level of CRP within 72 h of IFX infusion was reported as an effect on the cytokine profile including IL-6 [12]. At this time, it is not possible to explain if the reduction of CRP by IFX via an effect on the cytokine profile is directly related to the observed changes in the CRP (week 2/week 0) ratio. However, if we accept that the therapeutic efficacy of IFX in UC is via neutralization of TNF-α, then patients with UC may be subdivided into those in whom TNF-α has a dominant role in the exacerbation of the disease and those in whom TNF-α does not have a major role, bearing in mind that the exacerbation depends on other factors. If this assumption is validated by sound clinical data, it should contribute to better treatment of UC.

Hitherto, other investigators have attempted to identify predictors of clinical response to IFX in UC patients [23–25]. A low TNF-α mRNA expression in the colorectal mucosa at pre-treatment has been associated with better clinical and endoscopic remission rates following IFX induction therapy [23]. In contrast, patients who were seropositive for perinuclear anti-neutrophil cytoplasmic antibodies (pANCA) and were anti-saccharomyces cerevisiae antibodies negative (pANCA+/ASCA-) showed significantly lower clinical response to IFX [24]. Fasanmade, et al. [25] reported that blood concentration of albumin was associated with trough IFX levels and the response to IFX. We did investigate the relevance of blood albumin levels to IFX efficacy in our ROC model, but found that albumin level was not a sensitive predictor of response to IFX. However, unlike albumin, in patients with IBD, serum CRP level correlates well with disease activity and is the most widely monitored laboratory marker [9–11, 13]. Further, it has been reported that patients with UC or CD in whom CRP level decreased to normal range after completion of anti-TNF therapy had better remission maintenance time [14, 17, 18].

Unlike, TNF-α mRNA, and serum (pANCA+/ASCA-), measurement of CRP is an uncomplicated undertaking. However, the prediction of clinical response to therapy by the absolute value of CRP can be complicated due to the deviation of CRP values between different laboratories. Instead, application of the CRP ratio to judge clinical outcomes is more appropriate because CRP ratio is less affected by the differences between laboratories. The minimum detectable concentration of CRP in the method we used in this study is 0.01 mg/dL. Therefore, setting a cut-off value of 0.19 for the CRP (week 2/week 0) ratio means that patients with baseline CRP ≥ 0.05 mg/dL could be factored into the assessments. However, 5 of 72 patients had a baseline CRP value of <0.05 mg/dL. This means that the CRP (week 2/week 0) ratio will not work as the predictor for approximately 7 % of patients.

At this point, we should state specific limitations featured in this study. Firstly, the number of patients included was not large enough to allow showing stronger or otherwise weaker significance levels in our sub-group comparisons. Secondly, patients with baseline CRP value below 0.05 mg/dL could not be included in the assessment of the CRP as predictor of response to IFX. Thirdly, full endoscopy data was not included in our analyses of clinical outcomes.

## Conclusion

In this study, a significant difference in CRP between IFX responders, partial-responders and non-responders during IFX induction therapy was found 2 weeks following the first IFX infusion. The differences in CRP (week 2/week0) ratios between the responders, partial-responders and non-responders were more striking than anything we had expected. This was determined by the application of an ROC model. This finding is potentially interesting in clinical setting because monitoring the CRP (week 2/week 0) ratio at an early stage like week 2 during IFX induction therapy might provide an indication to stop futile anti-TNF therapy.

## Abbreviations

CRP: C-reactive protein
f-IFX: functional infliximab
IBD: Inflammatory bowel disease
IFX: Infliximab
IL: Interleukin
IQR: Interquartile range
pMayo: partial Mayo
ROC: Receiver operating characteristic
TNF-α: Tumour necrosis factor-alpha
UC: Ulcerative colitis
CD: Crohn’s disease
